# The Morphofunctional Effect of the Transplantation of Bone Marrow Stromal Cells and Predegenerated Peripheral Nerve in Chronic Paraplegic Rat Model via Spinal Cord Transection

**DOI:** 10.1155/2015/389520

**Published:** 2015-11-08

**Authors:** Vinnitsa Buzoianu-Anguiano, Sandra Orozco-Suárez, Elisa García-Vences, Sara Caballero-Chacón, Gabriel Guizar-Sahagún, Luis Chavez-Sanchez, Israel Grijalva

**Affiliations:** ^1^Medical Research Unit for Neurological Diseases, High Specialty Medical Unit Specialty Hospital, Centro Médico Nacional Siglo XXI, IMSS (Mexican Social Security Institute), Avenida Cuauhtémoc 330, Doctores, Cuauhtémoc, 06720 México, DF, Mexico; ^2^Scientific Unit, Faculty of Medicine, UNAM-INMEGEN (National Autonomous University of Mexico-National Institute of Genomic Medicine), Periférico Sur 4809, Arenal Tepepan, Tlalpan, 14610 México, DF, Mexico; ^3^Faculty of Health Sciences, Anáhuac México Norte, Avenida Universidad Anáhuac 46, Lomas Anáhuac, 52786 Huixquilucan, MEX, Mexico; ^4^Department of Physiology and Pharmacy, Faculty of Veterinary Medicine and Zootechnics, UNAM (National Autonomous University of Mexico), Circuito Exterior, Ciudad Universitaria, Coyoacán, 04510 México, DF, Mexico; ^5^Medical Research Unit for Immunology, High Specialty Medical Unit Pediatric Hospital, Centro Medico Nacional Siglo XXI, IMSS (Mexican Social Security Institute), Avenida Cuauhtémoc 330, Doctores, Cuauhtémoc, 06720 México, DF, Mexico

## Abstract

Functional recovery following spinal cord injury (SCI) is limited by poor axonal and cellular regeneration as well as the failure to replace damaged myelin. Employed separately, both the transplantation of the predegenerated peripheral nerve (PPN) and the transplantation of bone marrow stromal cells (BMSCs) have been shown to promote the regrowth and remyelination of the damaged central axons in SCI models of hemisection, transection, and contusion injury. With the aim to test the effects of the combined transplantation of PPN and BMSC on regrowth, remyelination, and locomotor function in an adult rat model of spinal cord (SC) transection, 39 Fischer 344 rats underwent SC transection at T9 level. Four weeks later they were randomly assigned to traumatic spinal cord injury (TSCI) without treatment, TSCI + Fibrin Glue (FG), TSCI + FG + PPN, and TSCI + FG + PPN + BMSCs. Eight weeks after, transplantation was carried out on immunofluorescence and electron microscope studies. The results showed greater axonal regrowth and remyelination in experimental groups TSCI + FG + PPN and TSCI + FG + PPN + BMSCs analyzed with GAP-43, neuritin, and myelin basic protein. It is concluded that the combined treatment of PPN and BMSCs is a favorable strategy for axonal regrowth and remyelination in a chronic SC transection model.

## 1. Introduction

Traumatic spinal cord injury (TSCI) causes permanent disability characterized by paralysis and loss of sensitivity as well as multiple metabolic and systemic alterations associated with the dysfunction of the autonomic nervous system [1].

Until now, there is no effective treatment for both acute and chronic SCI, despite several strategies that have been carried out to promote regeneration and improve function. Within these strategies, the use of tissue transplantation has been proposed. Due to its organized structure, the use of peripheral nerve acts as a physical guide via which axons are encouraged to grow [2]. They are also distally connected and act as a neuroprotector for the preserved spinal cord [2–6]. Furthermore, due to the action of the Schwann cells and macrophages stemming from the PPN, the regeneration and axonal remyelination are supported, since they are capable of secreting growth factors such as brain-derived neurotrophic factor (BDNF), nerve growth factor (NGF), neurotrophin-3 (NT-3), and granulocyte-macrophage colony-stimulating factor (GM-CSF) which promote neuronal survival [7–13].

Another strategy employed is the use of adult bone marrow stromal cells (BMSCs) since they have the capacity for self-renewal and differentiation. It has been demonstrated that the use of BMSCs in TSCI assists in modulating the central nervous system (CNS) environment to promote its repair and secreting anti-inflammatory and antiapoptotic molecules and growth factors, which promote axonal growth, immunomodulation, angiogenesis, remyelination, and protection from cell death caused by apoptosis [14]. They have been shown to have the capacity to differentiate into different neural linages both in vitro and in vivo, including neurons, astrocytes, oligodendrocytes, Schwann cells, and microglia [15, 16]. Furthermore, they promote axonal regrowth, remyelination, and the improvement of locomotor function, since they are capable of secreting growth factors such as BDNF, NT-3, VEGF, and bFGF [17–22].

As well as those previously mentioned, there are multiple strategies that have been observed to promote axonal regrowth and the reworking of the central nervous system (CNS) in mammals that have suffered a TSCI. However, none of these alone has been able to reestablish total functionality of the injured spinal cord (SC). As such, it is feasible to believe that the use of two of these in conjunction would produce greater functionality.

The objective of this study was to evaluate the morphological and functional effect of transplanting BMSCs and PPN into chronic paraplegic rat model that has undergone complete spinal cord transection. Our hypothesis was that the combination of PPN and BMSCs would give better results compared to those obtained from untreated rats or rats treated with individual transplants.

## 2. Materials and Methods

### 2.1. Experimental Design

This study was authorized by the Research and Ethics Committee of the Hospital de Especialidades, Centro Medico Nacional Siglo XXI, Instituto Mexicano del Seguro Social (IMSS); use, handling, and care of animals were carried out following the Official Mexican Standard (Norma Oficial Mexicana) NOM-062-ZOO-1999, which is supported on international standards. A total of 84 Fischer 344 rats were used, aged between 8–10 weeks and weighing between 200 and 220 g. For the TSCI and transplant procedures, 39 females were used having been divided into four random groups (immunofluorescence and histology: Control group: 7 animals; fibrin glue group: 8 animals; PPN group: 12 animals; PPN + BMSCs: 12 animals; electron microscopy: 3 animals per group), 25 males were used as sciatic nerve donors, and 20 males were used to obtain BMSCs.

### 2.2. Surgical Procedures and Transplant Preparation

#### 2.2.1. Spinal Cord Injury

In order to produce the spinal injury, the rats were anaesthetized by the intraperitoneal injection of a mixture of ketamine (70 mg/kg) and Xylazine (10 mg/kg). A laminectomy was carried out at T9 before a sagittal section was made to the dural sac on the dorsal side. A complete transection of the exposed spinal cord was carried out immediately using microsurgery scissors; finally, the surgical wound was stitched using layered closure.

#### 2.2.2. Preparing the Peripheral Nerve for Transplantation

Twenty-one days before the transplant, the peripheral nerve donor rats were anaesthetized before undergoing a complete transverse section of the sciatic nerve in the upper part of the thigh; the caudal stump of the sectioned nerve was fixed to the surrounding muscle with a 5-0 nylon suture. On the day of the transplant, the rat was anaesthetized to extract a segment of sciatic nerve distal to the cut of approximately 2 cm in length. The nerve fragment was placed in chilled isotonic saline solution until the time of transplantation.

#### 2.2.3. Preparing the Cells for Transplantation

The BMSCs were obtained from rats euthanized with an overdose of sodium pentobarbital. The bone marrow was obtained from both femurs and tibias using a 200 *μ*L micropipette and deposited in a 15 mL conical tube with culture medium (Dulbecco's Modified Eagle (DMEM) GIBCO). Following this, the sample was centrifuged at 1500 rpm for 7 minutes. The cells were then separated using a Ficoll (SIGMA) (3 mL) gradient centrifuged at 2000 rpm at 24°C for 30 minutes. The total number of nucleated cells obtained was quantified and 9 × 10^6^ cells were seeded into a 75 cm^2^ culture flask (in 5 mL of DMEM with 20% fetal bovine serum (FBS), from GIBCO), 1 mL of L-Glutamine (GIBCO), 5 mL of HEPES (SIGMA), and 1 mL of Penicillin-Streptomycin (GIBCO); they were then placed in a water-jacketed incubator at 37°C with 5% CO_2_ until the cells formed a fibroblast monolayer. Finally, the BMSCs were reseeded onto the fibroblast layer and maintained for two weeks until transplantation time.

#### 2.2.4. Phenotypic Classification of BMSCs

**Figure 1:**
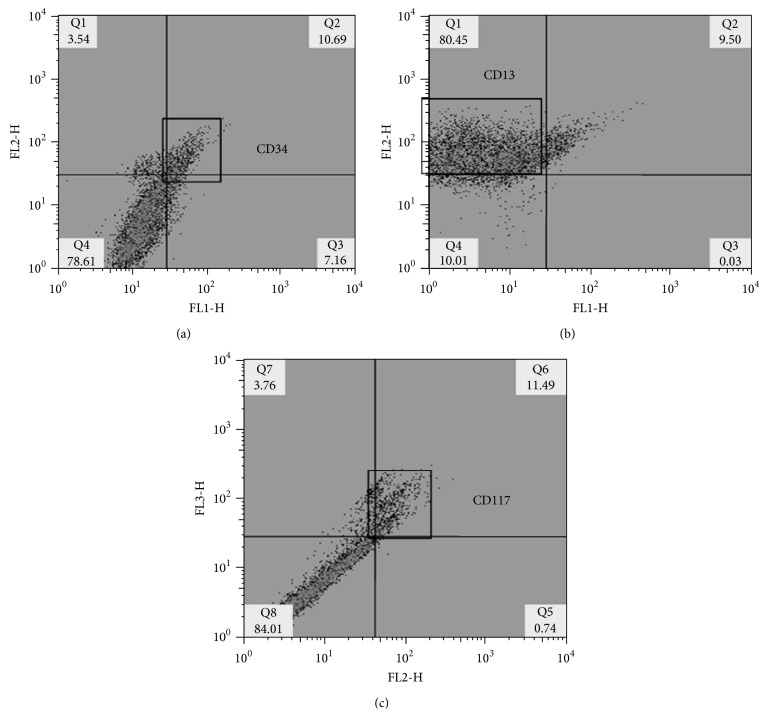
Bone marrow stromal cells (BMSCs) flow cytometry with Cd13 and Cd117. (a) Population of Cd34-positive cells (10.69%). (b) Population of Cd13-positive BMSCs (80.45%). (c) Population of Cd117-positive BMSCs (11.49%).

Flow cytometry was used to classify the mature BMSCs. The cells were centrifuged at 1500 rpm for five minutes and they were incubated with primary antibodies (Cd117 Millipore; Cd13 Santa Cruz Biotechnology Inc; Cd34 Santa Cruz Biotechnology Inc, all at a dilution of 1 : 100) in darkness for 20 minutes at 4°C. They were washed twice with FACS Buffer before being centrifuged again at 1500 rpm for five minutes. The cells marked with Cd117 were incubated in darkness for two hours with the secondary antibody (Alexa 488 or 586, Molecular Probes Invitrogen 1 : 200); they were then fixed in 4% paraformaldehyde for 1 hour before finally being quantified and analyzed with flow cytometry using the CellQuest Pro (BD Biosciences) program. Cell phenotype proportion is shown in Figure 1. 80.45% of the cells were positive for Cd13 (marker for subpopulation of mesenchymal stem cells); 11.49% were positive for Cd117 (marker for subpopulation of mesenchymal stem cells); and 10.69% of the cellular population was positive for Cd34 (specific control marker for hematopoietic stem cells). As such, the majority of cells transplanted were adult mesenchymal stem cells.

#### 2.2.5. Transplant

Four weeks after the spinal cord transection, the rats were assigned to one of four experimental groups. In Group 1 (Control, *n* = 7), the surgical wound was reopened enough to expose the dural sac. In Group 2 (positive Control *n* = 8), the dural sac was reopened and the scar was carefully removed from the spinal cord and the end of the medullary stumps, leaving a cavity of approximately 6 mm of amputated length; the cavity was filled with fibrin glue (BAXTER). In Group 3 (*n* = 12), the same procedure was carried out as described for group 2, except, in this case, 3 or 4 segments of the sciatic peripheral nerve, each of approximately 6 mm in length, were transplanted lengthways into the medullary cavity; the implants were fixed with fibrin glue (BAXTER). In Group 4 (*n* = 12), in addition to the procedures detailed for group 3, BMSCs were transplanted via four injections, two in the proximal medullary stump and two in the distal medullary stump, in the center of each hemicord; each injection contained 3 × 10^4^ cells in 5 *μ*L of Hank's solution (SIGMA).

### 2.3. Evaluations

#### 2.3.1. Motor Function Evaluation

Hindlimb locomotion was evaluated using the Basso, Beattie, Bresnahan (BBB) Locomotor Rating Scale [23]. The scale of the BBB evaluates the movements of the hindlimb of the animals; the scale oscillates between 0–21 points, where 0 is no movement and 21 is a normal movement of the hindlimbs. The evaluator was unaware of the treatment assigned to each animal. The animals were evaluated 24 hours after the transplant and every two weeks during the following 8 weeks.

#### 2.3.2. Processing of Samples for Immunofluorescence

Eight weeks after transplantation, the animals were anaesthetized with sodium pentobarbital (40 mg/kg i.p) before being perfused by intracardiac injection with 4% paraformaldehyde. A 2 cm long segment was obtained from the spinal cord with the injury zone in the center. The samples were placed in a 30% sucrose solution in PBS for 24 hours; 12 *μ*m thick sagittal frozen serial sections were then cut on a LEICA CM1510S cryostat. Following this, the sections were incubated for 48 hours at 4°C with the primary antibodies (Protein basic myelin (PBM) D18; Neuritin FL-142, and Gap-43 H-100, Santa Cruz Biotechnology Inc.). The sections were later washed with PBS and incubated for 2 hours with the secondary antibody (Alexa 488 Anti-Rabbit or Anti-Mouse, Molecular Probes Invitrogen); they were washed with PBS and stained using propidium iodide (red nuclei) for 1 minute. Finally, they were covered with Vectashield (Vector Labs) in order to be analyzed with a fluorescence microscope (Carl Zeiss). For each specimen, 6 photos were taken from the epicenter (transplant area) and the zones both proximal and distal to it. With the Image-Pro Plus 5.1 (Media Cybernetics) program, the intensity of green channel pixels corresponding to the Alexa 488 per area was quantified. A histogram, with previously calibrated intensity and spatial scale, was obtained from the intensity values contained within the image's bitmap to establish the intensity values such as the integrated intensity of each image. The optical density was determined in relation to the control group and expressed in pixels/mm^2^.

#### 2.3.3. Histology

The Kluver-Barrera staining method was performed on two specimens of each group; the sections were hydrated before being put into 95% alcohol; they were then placed in a Luxol Fast Blue solution overnight at 37°C. Following this, the excess colorant was removed using 95% alcohol and they were rinsed with distilled water; they were then washed with lithium carbonate and again rinsed with 70% alcohol and with distilled water. The sections were immediately placed in a purple Cresol solution for 6 minutes before being returned to the 70% alcohol and dehydrated with absolute alcohol. They were left to dry and fixed using Entellan synthetic resin (Merck). Finally, the samples were observed using a NIKON (Eclipse E600) microscope and the morphological changes were observed.

#### 2.3.4. Electron Microscope

The specimens were fixed with a Karnovsky solution (2.5% glutaraldehyde and 2% paraformaldehyde in a Sorensen solution) during 4 hours at 4°C. Following this, they were postfixed in 1% osmium tetroxide for 45 minutes at room temperature. The excess osmium tetroxide was removed by twice washing with distilled water for two minutes each time. They were dehydrated in alcohols of increasing concentration (50%, 70%, 95%, and 100%) to reach propylene oxide. Following dehydration, the tissue was included in Aldarite synthetic resin and was left to polymerize for 24 hours at 60 or 70°C. Once the blocks were carried out, semifine cuts were made to locate the zone to be evaluated before fine cuts were made that would be fixed to copper grids and stained with uranyl acetate and lead. The sections were analyzed with a Zeiss 906 transmission electron microscope and, for each group, 10 images were taken from the transition zone only between the PPN transplant and both the proximal and distal surrounding spinal cords in which only morphological changes were observed.

#### 2.3.5. Statistical Analysis

The Graph Prism 5.0 statistics program was used for descriptive analysis; measures of central tendency and statistical dispersion were used alongside tables and graphs. For statistical inference, a nonparametric ANOVA analysis test was used with Kruskal-Wallis ranges to determine the differences between groups, followed by a Mann-Whitney *U* test to identify the groups between which there was a difference. *p* < 0.05 was considered significant.

## 3. Results

### 3.1. Motor Function Results

**Figure 2:**
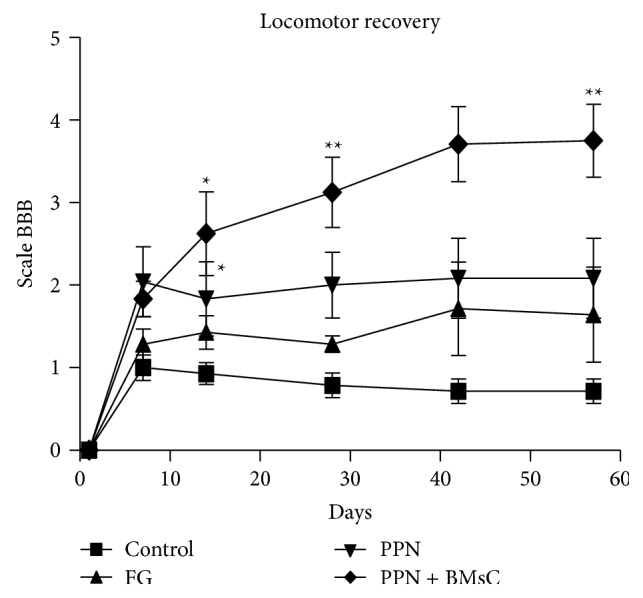
Analysis of locomotor function based on the BBB rating scale. *y*-axis corresponds to days of evaluation, where day 0 was the beginning of the evaluation within 24 hours after the transplant. The differences between the PPN and PPN + BMSCs groups can be observed when compared to the Control group (∗) (Kruskal-Wallis test, *p* < 0.05); furthermore, upon comparing the treatment groups, only the PPN + BMSCs group presented a significant difference from day 30 onwards compared to FG (∗∗) (Kruskal-Wallis test and Mann-Whitney *U* Test, *p* < 0.05 resp.), with no difference observed between the transplant groups. The graph represents the average ± standard error.

The hind extremity evaluation using the BBB rating scale showed severe motor function deterioration in the initial evaluation following the different experimental procedures. Scores improved marginally as time passed, especially for the PPN and BMSCs groups which reached an average rating close to 4 in contrast to the Control group which maintained a rating close to 1 (Figure 2).

### 3.2. Expression of GAP-43, Neuritin, and PBM: Microscopic Observations

**Figure 3:**
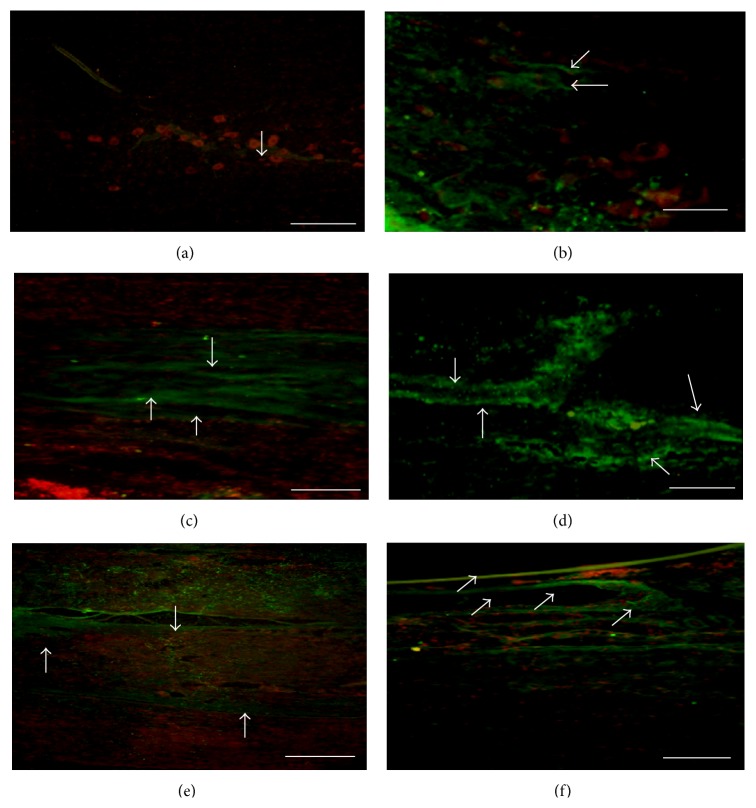
Photomicrography of the expression of GAP-43 in spinal cord. (a) Control group. (b) Fibrin glue group (FG). (c) PPN group, rostral zone of the SC. (d) PPN + BMSCs group, rostral zone of the SC. (e) PPN group, caudal zone of the SC. (f) PPN + BMSCs group, caudal zone of the SC. A greater quantity of GAP-43 positive fibers can be observed in panels (c) to (f) (white arrows, Alexa 488, green color), compared to panels (a) and (b). Furthermore, and easily visible, the positive fibers which were found in the PPN + BMSCs group ((e) and (f)) are thicker than those ones which were in the PPN group ((c) and (d)). All the tissue was contrasted with propidium iodide (nuclei red color). Calibration bar 20 *μ*m.

**Figure 4:**
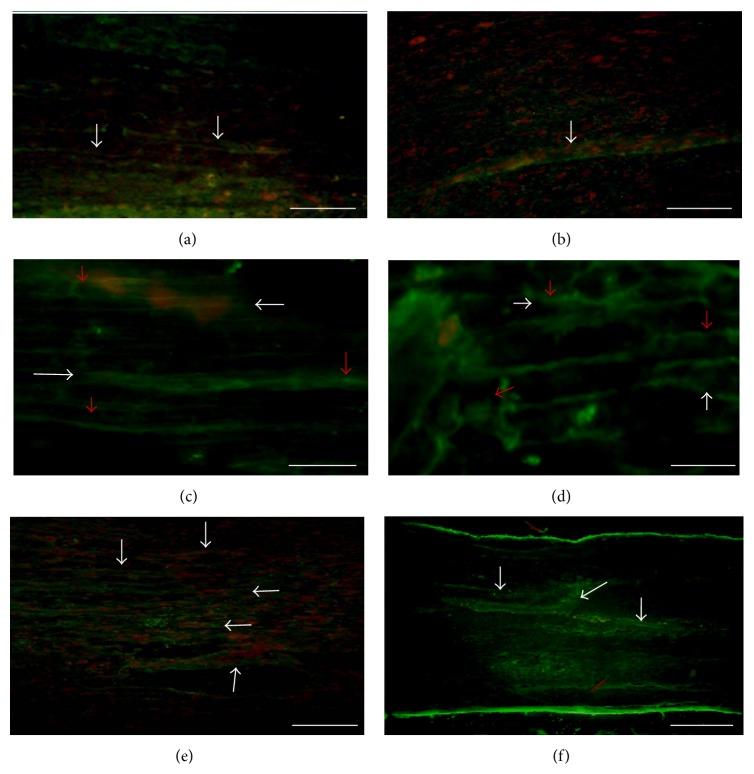
Photomicrography of the expression of Neuritin in spinal cord. (a) Control group. (b) Fibrin glue group (FG). (c) PPN group, rostral zone of the SC. (d) PPN + BMSCs group, rostral zone of the SC. (e) PPN group, caudal zone of the SC. (f) PPN + BMSCs group, caudal zone of the SC. A greater quantity of Neuritin positive fibers can be observed in panels (c) to (f) (white arrows, Alexa 488, green color), compared to panels (a) and (b). We can also observe that growth cones (red arrows) were only presented in the treated groups (panels (c) and (d)). All the tissue was contrasted with propidium iodide (nuclei red color). Calibration bar 20 *μ*m.

**Figure 5:**
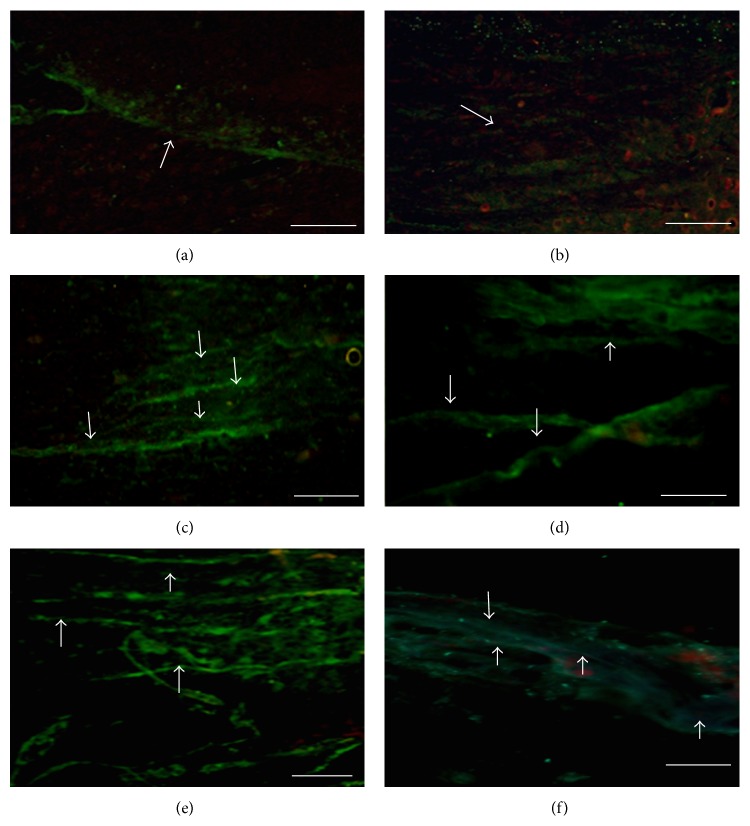
Photomicrography of the expression of Basic Protein Myelin in spinal cord. (a) Control group. (b) Fibrin glue group (FG). (c) PPN group, rostral zone of the SC. (d) PPN + BMSCs group, rostral zone of the SC. (e) PPN group, caudal zone of the SC. (f) PPN + BMSCs group, caudal zone of the SC. A greater quantity of PBM-positive fibers can be observed in panels (c) to (f) (white arrows, Alexa 488, green color) in contrast to panels (a) and (b). All the tissue was contrasted with propidium iodide (nuclei red color). Calibration bar 20 *μ*m.

In the qualitative evaluation of the histology images, a greater quantity of GAP-43 positive axons (Figure 3) and Neuritin was found in the transplant group compared to the Control group, in the zones both rostral and caudal to the injury (Figure 4). Furthermore, the PPN + BMSCs group axons were thicker than those observed in the PPN group (Figure 3). Finally, the presence of growth cones marked with Neuritin was only present in the PPN and PPN + BMSCs groups (Figure 4). A greater number of PBM-positive axons were also observed in the PPN and PPN + BMSCs groups in the zones rostral and caudal to the transplant (Figure 5).

### 3.3. Expression of GAP-43, Neuritin, and PBM: Quantification of Fluorescence Intensity

**Figure 6:**
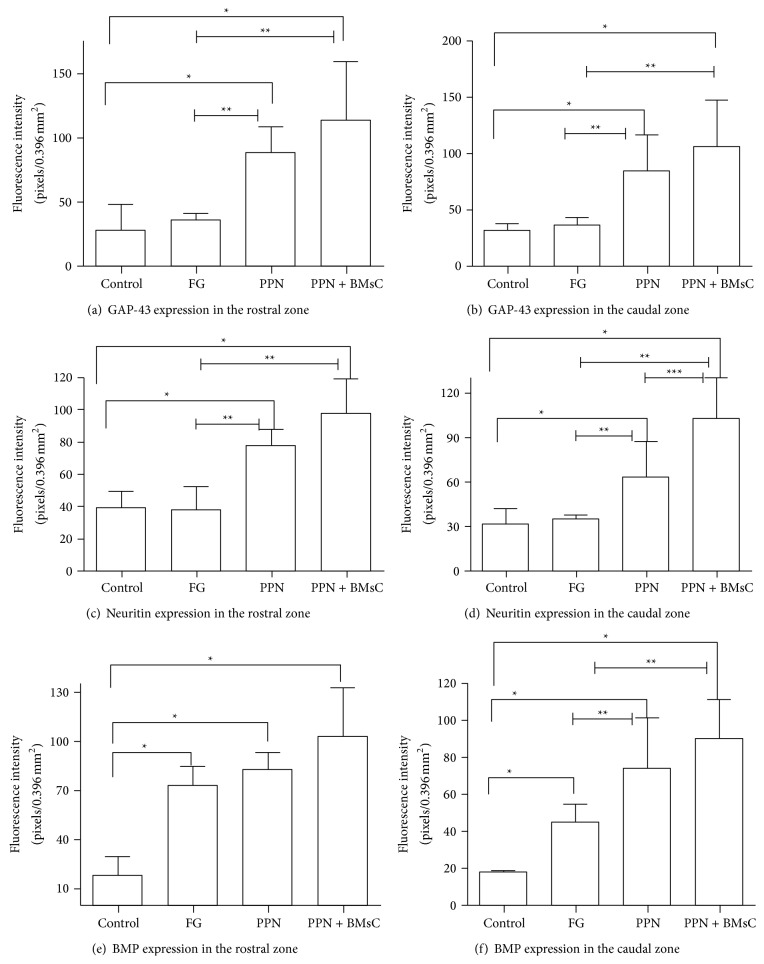
Fluorescence intensity analysis. Panels (a) and (b), expression of GAP-43: in both areas of the SC, a significant difference can be observed in the FG, PPN, and PPN + BMSCs groups compared to the Control group (Kruskal-Wallis test, *p* < 0.05) as well as a significant difference in the treatment groups compared to the FG group (Mann-Whitney *U* test, *p* < 0.05). Panels (c) and (d), expression of Neuritin: it can be observed that the treatment groups obtained a significant difference when they are compared to the Control and FG groups (Kruskal-Wallis test and Mann-Whitney *U* test, *p* < 0.05). Panels (e) and (f), expression of PBM in the SC: in both areas, a significant difference is observed between the treated groups and the Control and FG groups (Kruskal-Wallis test and Mann-Whitney *U* test, *p* < 0.05); furthermore, in the distal zone, a significant difference can be observed between the PPN + BMSCs group compared with the PPN group (Mann-Whitney *U* test, *p* < 0.05). ∗ is the difference with Kruskal-Wallis *p* < 0.05, and ∗∗ is the difference with *U*-MANN *p* < 0.05.

The GAP-43 fluorescence intensity was significantly greater in the groups that received a treatment (PPN + BMSCs, FG, and PPN) versus the Control group (*p* < 0.05) in both the rostral and caudal zones (Figures 6(a) and 6(b)); additionally, in the caudal zone (Figure 6(b)), the fluorescence intensity was significantly greater in the PPN + BMSCs and PPN groups versus FG (*p* < 0.05). The Neuritin fluorescence intensity (Figures 6(c) and 6(d)) and PBM (Figures 6(e) and 6(f)) in both the rostral and caudal zones was significantly greater in the PPN + BMSCs and PPN groups versus the FG and control groups (*p* < 0.05).

### 3.4. Electron Microscopy of Myelination

**Figure 7:**
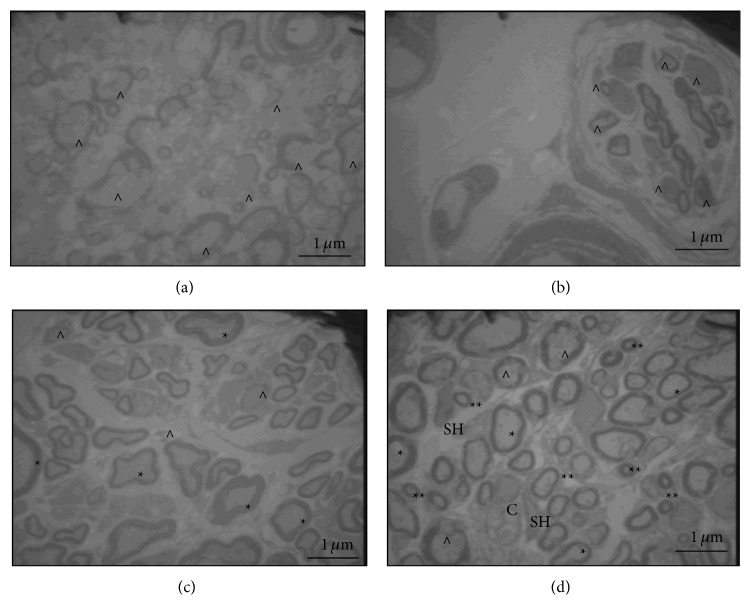
Electron photomicrography of distal stump to spinal cord. (a) Control; demyelinated axons and axons in process of demyelination (^∧^) (1600x). (b) FG; damaged axons (^∧^) (1600x). (c) PPN; myelinated axons (∗), macrophage close to a damage axon (MA), and damaged axons (^∧^) (1600x). (d) PPN + BMSCs myelinated axons (∗), newly formed axons (∗∗), some damaged axons (^∧^), and Schwann cell (SH) beside its axon and BMSCs (C) beside a Schwann cell (SH) (1600x). The images were taken with a transmission electron microscope LEO 906 E. Calibration bar 1 *μ*m.

**Figure 8:**
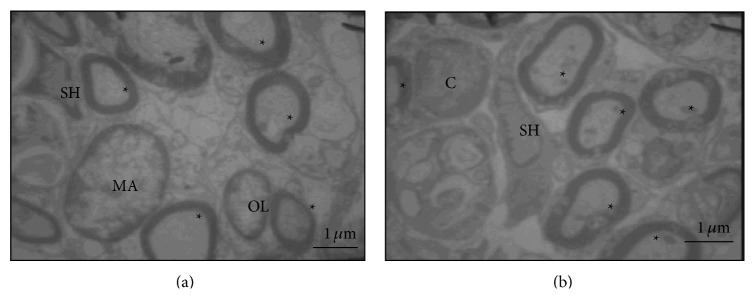
Electron photomicrography of distal stump to spinal cord. (a) PPN; Schwann cell (SH) interacts with a myelinated axon (∗); an oligodendrocyte (OL) can also be observed beside an axon, and a macrophage (MA) is seen in the left corner (1600x); (b) BMSC interacts (C) with a Schwann cell (SH) beside a myelinated axon (∗) (4646x). The images were taken with a transmission electron microscope LEO 906 E. Calibration bar 1 *μ*m.

In the PPN and PPN + BMSCs groups, it was observed that the axon myelin found had a well-defined and better-preserved structure; furthermore, there were a greater number of myelinated axons in contrast to Control and FG groups (Figure 7). Finally, in the PPN + BMSCs group, there were several thin axons rounded by a thin sheath of myelin and beside to a Schwann cell, which we consider the new axons (Figure 7). In both treatment groups, it was observed that the myelinated axons were ensheathed by the Schwann cells (Figure 7); on the other hand, in the PPN + BMSCs group, the BMSCs were found along with the axons and the Schwann cells (Figure 8).

### 3.5. Tissue Integration

**Figure 9:**
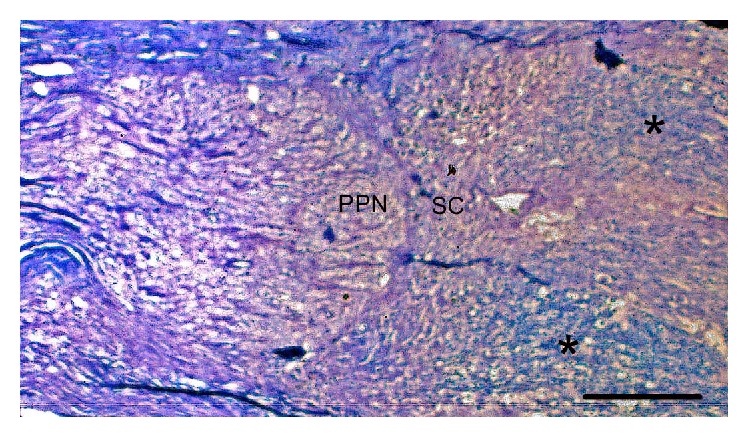
Spinal cord transplant interface. Photomicrography of a specimen from the PPN + BMSCs group in which excellent adhesion between the transplanted nerve (PPN) and the distal spinal cord (SC) stump can be observed. The spinal cord shows a good level of myelination (∗). Kluver-Barrera staining. Calibration bar 500 *μ*m.

From the samples analyzed with Luxol Fast Blue, it was observed that in the groups receiving PPN and PPN + BMSCs transplant, the surrounding spinal cord structure was adequately preserved and there was good acceptance between the transplanted PPN and the preserved SC (Figure 9).

## 4. Discussion

Functional recovery of the spinal cord following a traumatic injury with complete paralysis and secondary loss of sphincter control has been achieved using diverse therapeutic strategies in animal models. Nevertheless, therapeutic human trials carried out to date have had limited success. It is with that objective, therefore, that proposals for new alternatives using both single and combined treatment alternatives continue to be made.

It would appear that better results are achieved using combined treatments compared to single treatments. Using an acute model of complete transection, Guzen et al. [24] demonstrated that axonal regrowth and improved locomotor function took place following PPN transplantation; they also demonstrated that axonal regeneration and locomotor improvement increased when the PPN was combined with FGF-2, making it more effective than the use of PPN alone [24]. Using an acute model of complete transection, Koda et al. [25] also demonstrated that axonal regrowth and improved locomotor function occurred following the transplantation of BMSCs; however, the combination of BMSCs + BDNF showed greater effectiveness since it promoted a greater number of regenerated axons and increased locomotor function [25].

In this study, the therapy proposed to encourage axonal regrowth, remyelination, and functional recovery was the combined use of PPN and BMSC; considering that separately, each one of them has been shown to have a beneficial effect following the TSCI as previously mentioned.

The evaluation of axonal regrowth using the GAP-43 protein, known to be related to axonic fiber growth following a TSCI [26], showed a greater number of protein-positive fibers in the group transplanted with PPN than in Control group. Coinciding with Yuan et al. [27] in an axotomy model, GAP-43 positive fibers were found following the PPN transplant [27]. Following the PPN transplantation in an acute model of complete transection, Guzen et al. [24] also observed a greater quantity of GAP-43 positive fibers [24]. Furthermore, this study also observed that the PPN + BMSCs group had a greater number of GAP-43 positive fibers compared to the PPN group and they were also thicker than those of the PPN group. To date, no publications on the complete transection model are known to associate the use of BMSCs and the expression of GAP-43. However, Čížková et al. [28] observed the expression of GAP-43 following the transplantation of BMSCs in a compression model [28]. Finding GAP-43 positive fibers in another study using a contusion model, Kamada et al. [29] demonstrated that axonal regrowth was promoted following the transplantation of BMSCs [29]. The expression of GAP-43 might be mainly due to the PPN since it expresses different substances to support its regeneration such as trophic factors, adhesion molecules, and extracellular matrix molecules; [30] these factors provide a stimulating environment to facilitate the growth of CNS axons and support locomotor function [30]. However, since this expression is greater in the combined group, this could also be due to the contribution of the BMSCs given that the use of BMSCs in TSCI helps modulate the CNS environment to promote self-repair, secreting trophic substances that promote axonal growth [14].

As well as observing the presence of GAP-43, this study only found Neuritin immunoreactivity in the transplant groups PPN and PPN + BMSCs, indicative of the presence of growth cones; however, there was no significant difference between the transplant groups. Neuritin is the protein implicated in neuronal plasticity, relating to neurite growth [31]. There are no publications on complete section models in respect to this marker; however, Sarah Busch and colleagues have associated the presence of growth cones with the regrowth of sensory axons in a spinal cord compression model [32].

Myelination is essential to maintaining optimum function of the CNS since it promotes the conduction of nerve impulses and provides metabolic and trophic support; [33] so, when a TSCI occurs, the destruction of myelin affects motor and sensory function in the aforementioned manner. Structural affectation in different TSCI models can be identified by evaluating the level of myelination. PBM has been used to evaluate the level of myelination in different experimental models. In this study, a greater expression of PBM was observed in the transplant groups, especially in the distal segment of the spinal cord and mainly in the PPN + BMSCs group. Furthermore, in the electron microscope study, it was demonstrated that the PPN + BMSCs group had a greater quantity of myelinated axons which were more robust and which had ramifications. There are no studies known to evaluate the effect of PPN and BMSCs transplant on PBM in a chronic complete transection model. However, in an acute complete transection model, Chen et al. [34] demonstrated that they obtained a greater number of myelinated axons after transplanting BMSCs and the myelin had a uniform and more dense structure [34]. This property can be explained by the capability of the BMSCs to differentiate into myelinating cells. This is in line with the studies carried out by Akiyama et al. [35] who used a model of radiation injury, in which the myelinating cells were destroyed after transplantation of BMSCs, demonstrating that they promoted the myelination of bare axons which improved the nerve impulse [35].

Additionally, cells with a morphology that was different from that of the nervous tissue were observed near the myelinated axons; these cells could correspond to differentiated BMSCs, which can differentiate into myelinating cells as mentioned above [35], and, together with the Schwann cells resulting from the PPN, they help bring about a better myelination of regenerated axons as was demonstrated by Dam-Hieu et al. [36] in a model of hemicordotomy, in which the axons were myelinated by the actions of the Schwann cells following the transplantation of PPN [36].

Finally, and as a result of the beneficial mechanisms already mentioned in relation to both the PPN and the BMSCs, it was observed that, in the PPN and PPN + BMSCs transplant groups, the surrounding spinal cord structure was adequately preserved and that there was good acceptance with the PPN, which can be categorized as neuroprotection. This is in line with the study published by Guizar-sahagun et al. [3], in which they observed that the use of PPN acted like a shock absorber for substances produced by the secondary injury mechanisms, protecting the SC surrounding the injury zone, expressed as improved medullary tissue preservation [3]. On the other hand, Feng et al. [30] showed that, following the use of PPN in a model of contusion, there was greater neuronal preservation, which is due to the fact that the PPN promotes a microenvironment in which the Schwann cells secrete growth factors such as BDNF, NGF, and NT3 which improve neuronal survival [30]. It has been seen that the neuroprotective capacity of the BMSCs comes from the secretion of different substances, which can promote immunomodulation, repair, remyelination, axonal regrowth, and improved function. Using a model of compression, Quertainmont et al. [14] demonstrated that, in transplanting BMSCs, their neuroprotective effects came from the secretion of molecules such as the ciliary neurotrophic factor (CNT-F), the monocyte chemoattracting protein-1 (MCP-1), and the granulocyte-macrophage colony-stimulating factor (GM-CSF) which support the survival and differentiation of oligodendrocyte precursor cells, promoting the clearance of myelin debris and protecting the neurons and glial cells from apoptosis. They also observed an increase in the secretion of anti-inflammatory cytokines such as IFN-*γ* and IL-10 as well as the secretion of growth factors such as BDNF and NGF, which help protect the neurons during toxic events, promote regrowth, and repair and reorganize the neuronal connections and which also stimulate neurogenesis and protect tissue, decreasing scar formation. Also observed were the antiangiogenic effects of the secretion of the vascular endothelial growth factor (VEGF) [14].

Despite the fact that there were significant locomotion differences between transplanted and nontransplanted animals, the improvement was modest. On the other hand, although there was a tendency in favor of combined transplantation (PPN + BMSCs), the difference versus PPN group was not significant. In both cases, it can be due to the short term functional follow-up, because it has been seen that improvement begins at the third month after implemented treatment. Poor functional improvement was observed in models of complete transection that received no additional treatment, obtaining an average BBB rating scale score of 4 points [37]. Following transplantation of PPN in an acute model of complete transection, Guzen et al. [24] obtained an average score of 5 on the BBB rating scale eight weeks after treatment [24]. Following the transplantation of BMSCs in another acute injury study, Chen et al. [34] observed an average of 8 points on the same scale following eight weeks of treatment [34]. It is possible that there is additional functional improvement with long term follow-up as reported by Vaquero and Zurita in a severe contusion model in which the experimental animals achieved a 13 point score with BMSCs transplantation after a one-year follow-up [21].

## 5. Conclusions

The transplant of PPN + BMSCs in a chronic complete spinal cord transection model showed significantly great myelination in the preserved spinal cord rostral and caudal to the transplant area compared to PPN. However, although axonal regrowth and functionality were not significant, the PPN + BMSCs group showed high levels. The transplanted groups showed significantly greater axonal regrowth, myelination, and functionality than the Control groups. Additional studies are needed to evaluate the long term functional effect. The use of new combinations that potentially increase locomotor function in the same injury model used in this study is also proposed.
